# *In silico* comparison of protein-bound uremic toxin removal by hemodialysis, hemodiafiltration, membrane adsorption, and binding competition

**DOI:** 10.1038/s41598-018-37195-1

**Published:** 2019-01-29

**Authors:** Vaibhav Maheshwari, Stephan Thijssen, Xia Tao, Doris H. Fuertinger, Franz Kappel, Peter Kotanko

**Affiliations:** 1 Renal Research Institute, New York, USA; 2 Global Research & Development, Fresenius Medical Care, Bad Homburg, Germany; 3 Institute for Mathematics and Scientific Computing, University of Graz, Graz, Austria; 4 Icahn School of Medicine at Mount Sinai, New York, USA

## Abstract

Protein-bound uremic toxins (PBUTs) are poorly removed during hemodialysis (HD) due to their low free (dialyzable) plasma concentration. We compared PBUT removal between HD, hemodiafiltration (HDF), membrane adsorption, and PBUT displacement in HD. The latter involves infusing a binding competitor pre-dialyzer, which competes with PBUTs for their albumin binding sites and increases their free fraction. We used a mathematical model of PBUT/displacer kinetics in dialysis comprising a three-compartment patient model, an arterial/venous tube segment model, and a dialyzer model. Compared to HD, improvements in removal of prototypical PBUTs indoxyl sulfate (initial concentration 100 µM, 7% free) and p-cresyl sulfate (150 µM, 5% free) were: 5.5% and 6.4%, respectively, for pre-dilution HDF with 20 L replacement fluid; 8.1% and 9.1% for post-dilution HDF 20 L; 15.6% and 18.3% for pre-dilution HDF 60 L; 19.4% and 22.2% for complete membrane adsorption; 35.0% and 41.9% for displacement with tryptophan (2000 mg in 500 mL saline); 26.7% and 32.4% for displacement with ibuprofen (800 mg in 200 mL saline). Prolonged (one-month) use of tryptophan reduces the IS and pCS time-averaged concentration by 28.1% and 29.9%, respectively, compared to conventional HD. We conclude that competitive binding can be a pragmatic approach for improving PBUT removal.

## Introduction

Protein-bound uremic toxins (PBUTs) have been implicated in numerous deleterious effects in chronic kidney disease (CKD) patients as well as in end-stage renal disease (ESRD) patients^1^. In ESRD patients on hemodialysis (HD), there is a growing literature suggesting that improving the dialytic removal of these metabolites can improve the HD patients’ outcomes; however, PBUTs removal in standard high-flux HD is significantly smaller compared to removal of non-protein bound toxins^2^. Also, recent research indicated that frequent hemodialysis did not significantly lower levels of the putative uremic toxins p-cresyl sulfate (pCS) or indoxyl sulfate (IS)^3^. Fundamentally, the problem lies in their protein binding which reduces the free dialyzable fraction to such an extent that conventional high-flux HD provides only inadequate removal of PBUTs.

In HD patients, several PBUTs are found in excess, e.g. 3-carboxy-4-methyl-5-propyl-2-furanpropionate (CMPF), hippuric acid (HA), indole-3-acetic acid (IAA), indoxyl sulfate (IS), p-cresyl glucuronide (pCG), p-cresyl sulfate (pCS) etc., with protein-bound fraction in serum ranging from 30% to 99%^4^. Among all PBUTs, IS and pCS, both with protein-bound fraction >90%, are the most studied PBUTs^1^; both are often considered marker of this class of toxins^2^. Pre-dialysis concentration of IS and pCS have been found to be as much as 116-fold and 41-fold higher, respectively, than in the age-matched healthy controls, while concentrations of unbound marker toxins, urea and creatinine, were only 5- and 13-fold higher, respectively^5^. Both IS and pCS have been causally associated with pathophysiological events in HD patients such as cellular dysfunction, oxidative stress, cell senescence, to name a few^1^. IS interacts directly with macrophages and endothelial cells and accelerates atherosclerosis^6^, while pCS has proinflammatory effects on non-stimulated leucocytes^7^ and also damages osteoblastic cells through ROS production^8^. Typical reduction ratios of IS and pCS in a high-flux HD is less than 35%^4^, while the same for urea and creatinine is more than 70%, highlighting the inefficiency of conventional HD to remove PBUTs. Various methods for improving the dialytic removal of PBUT, such as hemodiafiltration^9^, membrane adsorption^10,11^, and competitive binding^12^ have been tested in patient population and in experimental setup. Comparison of all extracorporeal techniques in human subjects with appropriate power is practically infeasible; *in vitro* studies will also be very challenging, for example due to difficulties with simulating distribution volumes and liver metabolism. In this work, we provide an *in silico* comparative assessment of the effect of these methodologies on the PBUT removal.

To this end, we used a model developed by Maheshwari *et al*.; this model accounted for dynamic equilibrium between protein, toxin, and protein-toxin complex^13^. In this work, we expanded our earlier model of HD to account for the effect of (1) competitive binding among toxins and infused drug, (2) ideal membrane adsorption, and (3) pre- and post-dilution HDF on PBUTs removal. We focused our analysis on IS and pCS.

## Results

We used a model to mimic dialysis in a HD patient, with an initial plasma volume (*V*_*pl*_) of 3.5 L, 12 L of interstitial fluid (*V*_*is*_), 28 L of intracellular toxin distribution volume (*V*_*ic*_), and 35% hematocrit, using an Optiflux F180NR high-flux dialyzer surface area 1.8 m^2^. Dialyzer specifications are: housing diameter is 4 cm, comprising 12300 fibers, fiber inner radius is 105 µm, fiber wall thickness is 35 µm, and fiber length is 23 cm^13^. The calculated equilibrium association constants (*K*_A_) for IS and pCS were 3.64 × 10^4^ M^−1^ and 5.21 × 10^4^ M^−1^, respectively. These *K*_A_ values are in agreement with literature reported values for IS and pCS^14^. For different dialysis modalities, the reduction ratios, total removal, and toxin clearance for both IS and pCS are given in Table 1.

**Table 1:** Indoxyl sulfate (IS) and p-cresyl sulfate (pCS) reduction ratio (RR), net removal in 4 hour session, and dialytic clearance by various dialysis modalities.

DIALYSIS MODALITY		Indoxyl sulfate (IS)			p-cresyl sulfate (pCS)		
		RR [%]	Removal [mg]	Clearance [mL/min]	RR [%]	Removal [mg]	Clearance [mL/min]
Conventional hemodialysis (HD)		36	88.0	21.3	27	90.0	15.5
Pre-dilution HDF	Replacement fluid 20 L	39	92.9	23.0	30	95.7	16.8
	Replacement fluid 60 L	45	101.8	26.4	36	106.5	19.5
Post-dilution HDF (20 L)		41	95.1	23.9	32	98.2	17.5
Membrane adsorption (infinite *Q*_*d*_)		47	105.2	27.7	38	110.1	20.4
HD with displacer	Tryptophan (2000 mg)	57	118.8	34.3	48	127.7	25.7
	Ibuprofen (800 mg)	59	111.6	32.9	51	119.2	24.7

*Pre-dialysis concentrations for IS, pCS, and albumin were 100 µM, 150 µM, and 600 µM, respectively.

**Figure 1:**
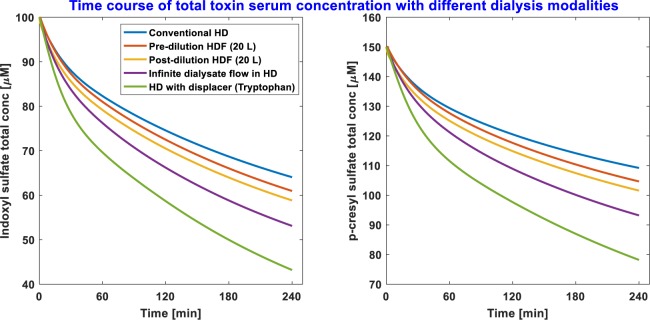
Indoxyl sulfate (IS) and p-cresyl sulfate (pCS) total concentration time course with different extracorporeal dialysis modalities. The line color codes are highlighted in the legend of the IS serum concentration profile.

The time course of total serum IS and pCS concentration in standard HD, pre-dilution HDF 20 L, post-dilution HDF 20 L, ideal membrane adsorption (infinite dialysate flow rate in standard HD), and standard HD with tryptophan as binding competitor, are given in Fig. 1. In HDF, pre-dilution HDF 60 L provided better IS and pCS removal when compared to that obtained by pre- or post-dilution HDF 20 L. For the same replacement fluid volume of 20 L, post-dilution HDF was superior to pre-dilution HDF (Table 1).

**Figure 2:**
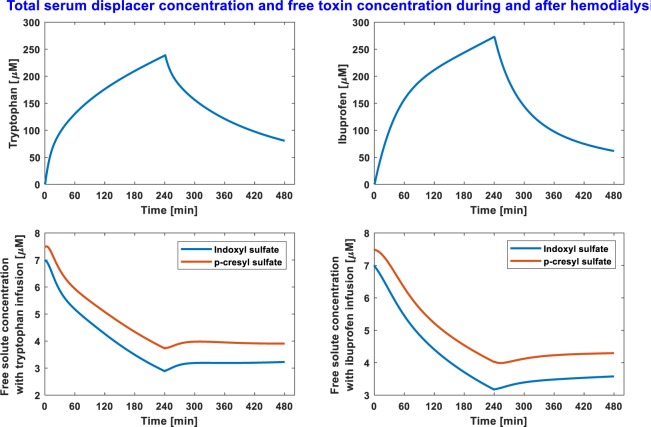
Serum binding-competitor concentration (top panels) and free toxin concentrations with binding-competitor infusion (bottom panels) during and after hemodialysis. The drug is infused at constant rate during 4 hour dialysis period. In the post-dialysis period (until next HD session) patient ingest the same amount of fluid at constant rate which is removed during 4-hour HD.

Tryptophan as binding competitor, infused at a constant rate during standard HD (2000 mg in 500 mL saline), provided the best removal of considered PBUTs. Tryptophan combined HD improved IS and pCS removal by 35% and 42%, respectively, over standard HD. The IS and pCS clearance in standard HD were 21.3 and 15.5 mL/min; since pCS is more strongly bound to albumin than IS, a lower clearance for pCS is expected. In tryptophan combined HD, the clearances for IS and pCS were also improved substantially by 61% and 65.5%, respectively. Similarly, ibuprofen combined HD (800 mg in 200 mL saline) outperformed all renal replacement therapies, except tryptophan combined HD. Toxin removal outcomes in ibuprofen combined standard HD can be found in Table 1. Note, while part of the infused drug is removed in the dialyzer itself during the first pass, a fraction of infused drug goes into the patient with venous return. Drug in the patient is continuously metabolized (tryptophan half-life is 2.83 hrs, ibuprofen half-life 2 hrs). At the end of dialysis session, total tryptophan mass in the patient was 432 mg (22% of total dose) or 306 mg of ibuprofen (38% of total dose). The competitor drug concentration profile during HD and post-HD is given in Fig. 2. We notice that serum drug concentration is reduced precipitously within 4 hours after HD. For respective infusion, the free solute concentration does not increase above the initial free solute concentration (Fig. 2).

**Figure 3:**
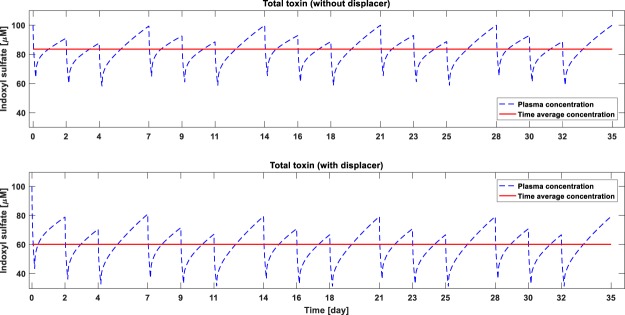
Monthly time-course of indoxyl sulfate (IS) concentration without (top panel) and with displacer (bottom panel). Toxin generation rate is assumed constant at 0.02477 mg/min during the simulated time-course. Intra-dialytic fluid removal and inter-dialysis fluid intake is kept constant at 2.4 L.

**Figure 4:**
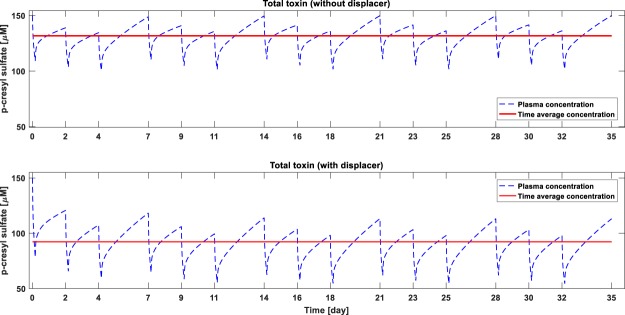
Monthly time-course of p-cresyl sulfate (pCS) concentration without (top panel) and with displacer (bottom panel). pCS generation rate is assumed constant at 0.02557 mg/min during the simulated time-course. Intra-dialytic fluid removal and inter-dialysis fluid intake is kept constant at 2.4 L.

**Figure 5:**
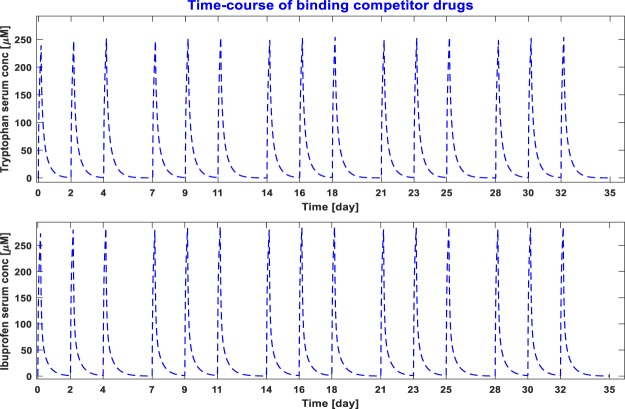
Monthly time-course of competitor drug concentration – tryptophan (top panel) and ibuprofen (bottom panel). Intra-dialytic fluid removal and inter-dialysis fluid intake is kept constant at 2.4 L. For tryptophan, endogenous production is not considered.

Since, tryptophan infusion during HD significantly outperforms other dialysis modalities including ibuprofen infusion during HD (Table 1), we investigated the long-term effect of tryptophan infusion. To study the long-term kinetics, the toxin generation rate is assumed constant for the simulation period of one month. Also, the intra-dialytic fluid removal was the same as the inter-dialytic fluid gain; both were set at 2.4 L. The saw-tooth concentration profile and time-averaged concentration (TAC) in standard HD and in tryptophan combined HD are given in Figs 3 and 4, respectively for IS and pCS. The TAC of IS reduced from 83.6 mg/L in standard HD to 60 mg/L in tryptophan combined HD (≈28% reduction), while TAC for pCS reduced from 132 mg/L to 92 mg/L (≈30% reduction). Since much of the infused drug is metabolized within few hours after dialysis, the drug concentration before the next dialysis session is zero (Fig. 5).

## Discussion

We employed a model simulation-based approach to compare different extracorporeal epuration modalities for PBUT removal. Our model simulations suggest that binding competition in HD extracorporeal circuit substantially improves the PBUTs removal over conventional HD, HDF, and membrane adsorption. This is the first report to provide a comparative assessment of PBUTs removal in standard HD, pre-dilution HDF 20 L and 60 L, post-dilution HDF 20 L, ideal membrane adsorption, and binding competition in HD where competitor drugs were tryptophan or ibuprofen. The model used in the current work is an extension of a previously reported model of PBUTs which was validated against clinical data^13^.

PBUTs have gained significant attention within nephrology community in recent years – for two reasons: (1) their association with mortality and morbidity of HD patients^1,15,16^, and (2) their insignificant removal due to strong protein binding. The dialytic removal of solutes primarily occurs by diffusive gradient across dialyzer membrane. Strong protein-binding results in very small free toxins concentration, leading to smaller diffusive gradient between blood and dialysate and thus small removal. Convection due to excess fluid removal during HD also contributes toward total PBUTs removal; however convective removal also depends on the free concentration of PBUTs.

Our model simulations of standard high-flux HD, for the typical initial concentrations in an HD patient, IS 100 µM (21.3 mg/L) and pCS 150 µM (28.2 mg/L), resulted in 36% and 27% reduction ratios; 21.3 mL/min and 15.5 mL/min clearance, and total toxin removal 88 mg and 90 mg, respectively for IS and pCS. There exists a variability in reported RR of IS and pCS, e.g., Deltombe *et al*. reported average RR of 37% for IS and 30% for pCS (10 patients)^17^; Lessafer *et al*. reported RR of 34.4 ± 17.3% for IS and 32.0 ± 18.8% for p-Cresol (10 patients)^18^; Martinez *et al*. reported RR of 30 ± 7% for indican and 20 ± 9% for pCS (5 HD patients)^19^. This variability in RR can be due to numerous factors such as, initial PBUT concentration, available free fraction at the start of dialysis, nutritional status affecting albumin concentration, type of dialyzer used in study, ultrafiltration volume, etc. The reduction ratios and removal obtained in our model simulations are typical for IS and pCS^4,9^. This model also captures the increase in protein bound fraction during HD and across the dialyzer, as observed *in vivo* by Deltombe *et al*.^17^; this has been illustrated previously^13^.

Since the removal of PBUTs is so poor in conventional HD, nephrologists investigated if convection in HDF, which has already shown its superiority over HD for middle-sized toxin removal^20^, may augment the PBUT removal. HDF is primarily operated in two modes: pre-dilution and post-dilution. Pre-dilution HDF dilutes the blood in arterial tube-segment (pre-dialyzer) and all the replacement fluid is removed along the fiber length. Though pre-dilution HDF reduces the free toxin concentration in inlet blood stream (due to dilution), toxin free fraction at dialyzer inlet will increase. This can be understood from the free fraction definition. In Equation (1), dilution of arterial blood will reduce the free protein concentration, resulting in increased free fraction ( *f* ); *K*_A_ is the fundamental property and remains constant. In effect, there are two contradictory factors induced by dilution of blood stream: (1) reduced free toxin concentration, (2) increased free fraction of solutes.

1 $$f=\frac{T}{T+PT}=\frac{1}{1+{K}_{A}\cdot P}$$

On the other hand, in the post-dilution HDF, extra fluid is removed along dialyzer length and replacement fluid is added post-dialyzer to compensate for excess fluid removed. In the post-dilution HDF, the free toxin fraction will reduce along the fiber because free protein concentration continuously increases due to removal of replacement fluid^13,17^, but free concentration will continuously increase due to hemoconcentration. Intuitively, it is difficult to predict the superiority of pre-dilution HDF over post-dilution HDF, or vice-versa. Bammens *et al*. were the first to delineate the PBUT removal advantage of one HDF mode over another^9^. In 14 stable HD patient cohort, they observed that post-dilution HDF provides better p-cresol clearance over pre-dilution HDF, when replacement fluid volume was 20 L. However, unlike the post-dilution HDF, the pre-dilution HDF can be performed with much higher replacement fluid volume, and Bammens *et al*. found that pre-dilution HDF 60 L outperformed post-dilution HDF 20 L. We also simulated all the HDF scenarios studied by Bammens *et al*. For the initial pCS concertation of 150 µM (28.2 mg/L) and 5% free, the pre-dilution HDF 20 L improved the total pCS removal by 6.4%, post-dilution HDF 20 L by 9.1%, and pre-dilution HDF 60 L by 18.3%; comparisons are made with respect to standard HD. Our model predictions are in agreement with the *in vivo* findings of Bammens *et al*.^9^, and indicates an excellent model fidelity. Note that in Bammens *et al*., despite the improved clearance in post-dilution HDF 20 L over pre-dilution HDF 20 L, removal was slightly better in the latter; nevertheless, only clearance comparison was statistically significant^9^. Comparable results were obtained for IS also (Table 1).

Though HDF slightly improves PBUT removal over standard HD, HDF is largely prescribed for enhancing middle-sized toxin removal, with 20–30% reduction in serum levels of β_2_-microglobumin in long-term^21^. It may be because unlike middle-sized molecules, PBUTs removal in HDF was not significant improvement over conventional HD^9^. Meert *et al*. compared post-dilution HDF and pre-dilution HDF but could not establish the superiority of one HDF mode over the other^22^. Similarly, Krieter *et al*., in a pilot study of 8 patients, also found comparable decrease of IS and pCS in HD and HDF^23^. Though pre-dilution HDF 60 L does provide significantly improved removal of both IS and pCS, it should be noted here that HDF with large replacement fluid is cost prohibitive, which may deter the dialysis providers from using convection based renal replacement therapies.

Adsorption of free toxins on the membrane surface is another approach investigated. In this approach, the free toxins are adsorbed on the adsorbents, impregnated in the dialyzer membrane^24^. Such membranes are known as mixed-matrix membrane (MMM). Tijink *et al*. provided significant improvement in PBUT removal using activated carbon impregnated MMMs *in vitro*; and anticipate that developed MMMs would suffice to remove the daily production of PBUTs^11^. Will these *in vitro* results translate into improved toxin removal *in vivo* can only be found out from clinical data. Adsorption of free solutes maintains high concentration gradient between blood and dialysate. In ideal scenario, adsorption technique can be considered equivalent to hypothetical infinite dialysate flow which will result in zero toxin concentration in the dialysate i.e. all toxins are adsorbed on the membrane surface. Without modeling the adsorption kinetics, we simulated the ideal adsorptive removal of PBUT by assuming infinite dialysate flow rate in standard HD. Compared to standard HD, this hypothetical membrane adsorption HD improved the single-session IS and pCS removal by 19% and 22%, respectively. Model simulations suggest that at its very best, membrane adsorption is close to pre-dilution HDF 60 L (Table 1). Here, we assumed that MMM specifications are same as that of the conventional high-flux dialyzer membrane. However, MMMs used in Tijink *et al*. are much thicker compared to conventional high-flux membrane: outer diameter ~1000 µm for MMM^11^ vs. 280 µm conventional high-flux dialyzer membrane in F180NR dialyzer^25^. Due to larger thickness of MMMs, significantly less number of hollow fibers will fit in the high-flux dialyzer casing or less mass transfer area will be available for unbound fraction of PBUTs. This will reduce the efficiency of MMMs. Pavlenko *et al*. recently developed new low flux MMMs with reduced fiber thickness^26^.

However, even the best MMMs with thickness same as conventional HD membrane may not provide the removal outcome we obtained in our simulations because toxin concentration in dialysate can never be zero, as assumed in the ideal adsorption. Some toxin will inadvertently diffuse in the dialysate side. Non-zero concentration in dialysate will reduce the concentration gradient between blood and dialysate, which will reduce the removal efficacy of MMMs.

Another attractive approach for PBUT removal is the use of binding competitors infused in the extracorporeal circuit. Xia *et al*. published the first report on efficacy of binding competitors for removal of PBUTs. In their *in vitro* single pass dialysis set-up, they observed 2.9-fold and 1.4-fold increase in IS removal using ibuprofen and tryptophan, respectively; this improvement is reported across dialyzer^12^. Important questions are: Is the competitive binding approach as efficient *in vivo* as it was *in vitro*? How much improvement will the competitive binding augmented HD elicit over standard HD? To answer these questions, we simulated two competitor drugs infusion, each infused in a conventional HD extracorporeal circuit pre-dialyzer: (1) ibuprofen (800 mg in 200 mL saline), (2) tryptophan (2000 mg in 500 mL saline). The drug dosages were within the FDA guidelines. For healthy subjects, 800 mg ibuprofen can be given every 6 hours, while tryptophan, an amino acid is often used in the management of neuropsychiatric disorders^27^, and may be safely prescribed in HD patients.

The choice of competitor drug is an important consideration: (1) the drug should be able to compete for toxin binding sites on albumin, (2) the drug should have a short half-life so that a patient eliminates the drug in very short time, (3) the drug should essentially be metabolized or cleared by organs other than the kidney, (4) a single drug molecule should be able to target multiple PBUTs, and (5) the drug and its metabolites should be benign, and moreover, provide salutary benefits to patient. Ibuprofen used in our analysis satisfies first four criteria; apparently, it is not benign for HD patients. Ibuprofen can potentially deteriorate the residual renal function and may cause gastrointestinal bleeding in few patients^28^. Tryptophan, on the other hand, satisfies all 5 criteria, but it has weaker albumin binding affinity compared to studied PBUTs. Despite the smaller binding affinity of tryptophan compared to IS and pCS, tryptophan augmented HD significantly improved the removal of both PBUTs. A question may arise here – though tryptophan has weaker albumin binding affinity (1.73 × 10^4^ M^−1^) compared to IS (3.64 × 10^4^ M^−1^) and pCS (5.21 × 10^4^ M^−1^), how does it compete with the toxins having higher binding affinity. To answer this question, we once again resort to free fraction definition (Equation (1)), which is dependent on free protein concentration ‘*P*’. Infusion of competitor drug molecule decreases the free protein concentration. The higher the drug dose (of tryptophan) or higher the binding affinity (of ibuprofen), the lower the free protein concentration, as in $P={P}_{tot}-P{T}_{1}-P{T}_{2}-PD$. A reduced free protein concentration results in higher free fraction of toxins. On a side note, it may be attractive and simpler to infuse competitor drugs before starting the dialysis session, but this approach may result in sharp increase in systemic free concentration of PBUTs pre-HD. Though short-term, this approach may accentuate the deleterious effects of PBUTs, since it is the free concentration of PBUTs which exerts the toxicity.

Though ibuprofen has stronger binding affinity towards albumin, tryptophan with weaker affinity provides better removal. One of the main reason for this is much larger dose of tryptophan (2000 mg) when compared to ibuprofen (800 mg). Interestingly, ibuprofen provided better reduction ratio, but smaller removal when compared to that obtained by tryptophan (Table 1). This is primarily due to very strong binding affinity of ibuprofen, which binds to albumin so strongly that only a very small amount of free ibuprofen is diffused in interstitial compartment, where larger reserve of PBUTs exist (due to higher amount of albumin). Tryptophan on the other hand, due to weaker binding affinity, easily diffuses in the interstitial pool and competes with toxins in there. This free toxin diffuses from interstitial pool to plasma, and subsequently goes in the extracorporeal circuit to end up in dialysate.

PBUTs are primarily bound to the most abundant protein in plasma, albumin, at different binding domains with different binding affinities. In the model presented here, we have neglected the binding contribution by secondary binding site, since more than 90% of IS and pCS is bound on primary binding site^14^; the same is assumed for competitor drugs, ibuprofen and tryptophan. Note that λ in free drug mass balance in Equation (2) accounts for first order elimination of drug by liver. Left-over drug in the patient can be one of the issue with competitive binding approach where HD patient may not clear the drug and its metabolites owing to absence of renal function. In such case, the lingering effects of competitor drug and metabolites can be detrimental to patient. This issue can be avoided by using drugs which not only work as competitor to PBUTs but also elicit salutatory benefits to HD patients. As mentioned earlier, ibuprofen cannot be recommended for chronic use in HD patients. Besides the risk of an adverse effect on residual renal function, ibuprofen may also inhibit tubular secretion of PBUTs in case there is still residual renal function, since Ibuprofen is a strong inhibitor of OAT1^29^. This may attenuate the beneficial effect of competitive albumin binding on PBUT removal. Fortunately, tryptophan does provide salutary effects to HD patients and may alleviate the sleep disorders in HD patients. A concern with chronic tryptophan use may be its metabolites (IS being one of them), which are toxic. However, in a cohort of 46 patients with CKD and HD, tryptophan intake based on 24-hr dietary recall was not correlated to IS plasma levels^30^. Note that, the majority of free tryptophan undergoes oxidative metabolism along the kynurenine pathway yielding kynurenines^31^. Kynurenines are also uremic toxins that are protein bound and are implicated in comorbid atherosclerosis by activating oxidative stress and leukocyte activation in endothelial and vascular smooth muscle cells and in neuropsychiatric symptoms.

Our simulations suggest that competitive binding can significantly improve the PBUT removal. More importantly, it changes the way we have perceived extracorporeal dialysis in the past. Though hemodialysis practice has improved dramatically since its inception in 1970, it has fundamentally remained the same in past 5 decades – a passive mass transfer process. Dialysis, essentially a diffusion-convection process, only mimics the latter part of kidney function, i.e., filtration. The other important function of native kidney, tubular secretion, is completely absent in the present hollow fiber membranes. Tubular secretion is modulated by presence of organic anion transporters (OATs), which in simplistic form, bind the free fraction of toxins/drugs and transport them from blood side to proximal convoluted tubule, from where these toxins end-up in urine. One may state that albumin and OATs compete for free fraction of these toxins/drugs. The reversal of this approach can be multiple substances competing for the same binding site on albumin so that free toxins can be removed by glomerulus or dialyzer membrane. The later approach is validated by Xia *et al*. 2016 and showed improved dialytic removal of IS and IAA *in vitro* with ibuprofen, furosemide, and tryptophan. Our model simulations reinforce these findings. Interestingly, binding competition is ubiquitous in pharmacokinetics literature where drug clearance and/or efficacy dramatically changes due to presence of other drug(s) competing for same binding sites on albumin^32^.

Unlike hemodiafiltration and membrane adsorption, competitive binding approach seems toxin specific. Though we focused on IS and pCS for analysis, the competitive binding methodology should be applicable for all PBUTs, subjected to the condition that both drug and toxin(s) share the same binding site on albumin, as in the case of IS, pCS, and tryptophan/ibuprofen. For toxins bound on another binding domain (Sudlow site I), a different competitor drug, such as aspirin, should be infused. To improve the removal of all PBUTs, a competitor drugs cocktail may be used, e.g. tryptophan/ibuprofen for Sudlow site II and aspirin for Sudlow site I. These competitor drugs can be infused together or separately, at constant rate or different rate, to maximize the removal of PBUTs.

In conclusion, we provided a comparative assessment of PBUT removal by modeling state-of-the-art hemodialysis techniques, namely, high-flux dialysis, pre- and post-dilution hemodiafiltration, membrane adsorption, and competitive binding. Our results show that tryptophan augmented HD provided the best removal outcome for both IS and pCS. The proposed model provides a mean to test the efficacy of novel competitor drugs which not only enhance the removal of PBUTs but may also provide salutary benefits to dialysis patients. We conclude that completive binding in HD should be considered as a clinically viable approach for improving dialytic removal of PBUTs.

## Materials and Methods

### Mathematical model

**Figure 6:**
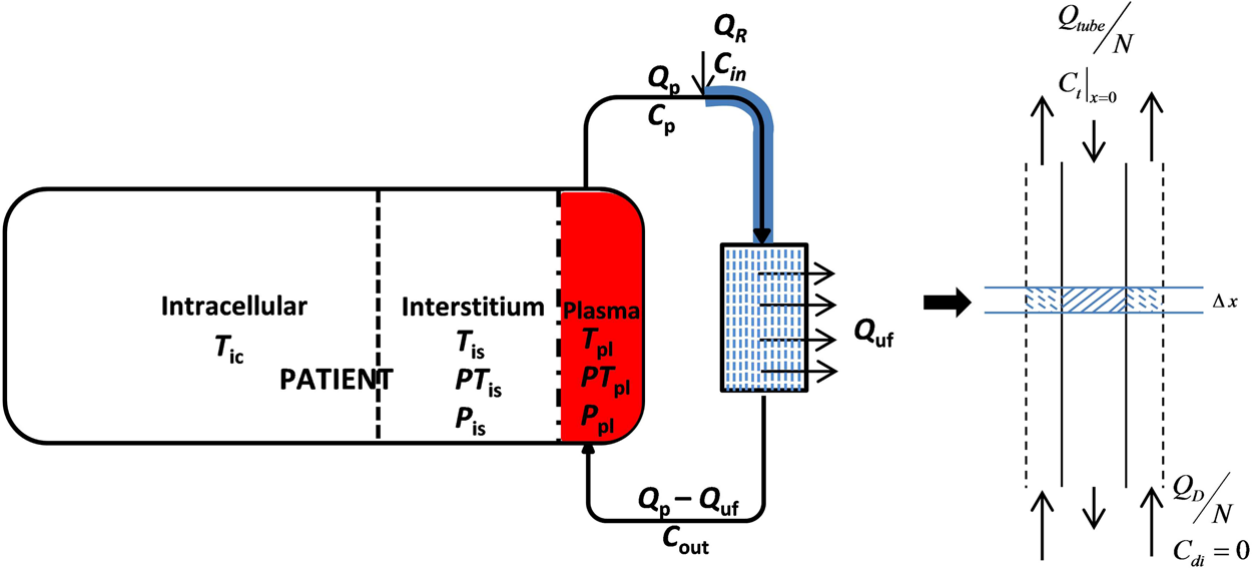
Block diagram of hemodialysis model system with drug competitor/pre-dilution replacement fluid being infused in arterial line. In three-compartment patient model symbols T, PT, and P denotes concentration of free solute, bound solute, and free protein. Here solute stands for both toxins IS, pCS, and displacer molecule tryptophan or ibuprofen. The subscript pl, is, and ic denote plasma, interstitial pool, and intracellular compartment, respectively. Shaded arterial tube segment is not applicable for post-dilution HDF, where a venous tube segment model was used instead. A single fiber is magnified to depict the flows in and around fiber.

Our original mathematical model of PBUTs dynamics comprised a three-compartment patient model and a spatiotemporal representation of the dialyzer^13^. The model was calibrated and validated using clinical data. In this work, we modified that model to account for the shift in binding equilibrium due to HDF replacement fluid dilution or due to binding competition between IS, pCS, and a competitor drug. The presented dialysis model system comprises three sub-models: (1) a three-compartment patient model, (2) an arterial tube segment model (for pre-dilution HDF and/or binding competition) or a venous tube segment model (for post-dilution HDF), and (3) a dialyzer model. A block diagram of the complete model is shown in Fig. 6. In the following, we provide details of each sub-model with corresponding model assumptions.

#### Patient model

The patient model consists of 3 compartments: plasma pool, interstitial pool, and intracellular pool. The free toxin is equilibrated among all three compartments, while the albumin-bound toxin is distributed in plasma and interstitial pool only. In the model presented here, toxin mass balance in the plasma compartment is illustrated below.

$${\rm{Rate}}\,{\rm{of}}\,{\rm{toxin}}\,{\rm{accumulation}}=-\,{\rm{Toxin}}\,{\rm{leaving}}\,{\rm{plasma}}\,{\rm{compartment}}\,\mathrm{with}({Q}_{p})+{\rm{Toxin}}\,{\rm{entering}}\,{\rm{plasma}}\,{\rm{compartment}}\,{\rm{with}}({Q}_{p}-{Q}_{uf})+{\rm{Diffusive}}\,{\rm{mass}}\,{\rm{transfer}}\,{\rm{from}}\,{\rm{interstitial}}\,{\rm{space}}+{\rm{Convective}}\,{\rm{mass}}\,{\rm{transfer}}\,{\rm{from}}\,{\rm{interstitial}}\,{\rm{space}}+{\rm{Reaction}}\,{\rm{rate}}\,{\rm{terms}}\,{\rm{based}}\,{\rm{on}}\,\mathrm{first} \mbox{-} \mathrm{order}\,{\rm{kinetics}}$$

where, *Q*_*p*_ and *Q*_*uf*_ are the plasma flow rate and the ultrafiltration rate, respectively. The resulting mass balances for free solutes, the protein-solute complexes, and free protein (protein without bound solutes) in the plasma compartment are shown in Equation (2). The protein and protein-solute complex exchange by lymphatic flow is neglected during HD. The protein-solute reaction kinetics is governed by the Law of Mass Action^33^. As for the solutes, we consider IS, pCS, and a competitor drug, denoted as *T*_1_, *T*_2_, and *D*, respectively. A description of all symbols and parameters can be found in Table 2. The reaction scheme looks as follows.

$$\begin{array}{c}P+{T}_{1}\mathop{\iff }\limits^{{K}_{A,{T}_{1}}\,}P{T}_{1};{K}_{A,{T}_{1}}=\frac{{a}_{1}}{{a}_{2}}\\ P+{T}_{2}\mathop{\iff }\limits^{{K}_{A,{T}_{2}}\,}P{T}_{2};{K}_{A,{T}_{2}}=\frac{{b}_{1}}{{b}_{2}}\\ P+D\mathop{\iff }\limits^{{K}_{A,D}\,}PD;{K}_{A,D}=\frac{{d}_{1}}{{d}_{2}}\end{array}$$

2 $$\begin{array}{ccc}\frac{d({V}_{pl}{T}_{1,pl})}{dt} & = & \begin{array}{c}-{Q}_{pi}{T}_{1,pl}+({Q}_{pi}-{Q}_{uf}){T}_{1,out}+{K}_{ip}({T}_{1,is}-{T}_{1,pl})+\frac{{V}_{is}}{{V}_{ex}}{Q}_{uf}{T}_{1,is}\\ +(-{a}_{1}{T}_{1,pl}{P}_{pl}+{a}_{2}P{T}_{1,pl}){V}_{pl},\end{array}\\ \frac{d({V}_{pl}P{T}_{1,pl})}{dt} & = & -{Q}_{pi}P{T}_{1,pl}+({Q}_{pi}-{Q}_{uf})P{T}_{1,out}+({a}_{1}{T}_{1,pl}{P}_{pl}-{a}_{2}P{T}_{1,pl}){V}_{pl},\\ \frac{d({V}_{pl}{T}_{2,pl})}{dt} & = & \begin{array}{c}-{Q}_{pi}{T}_{2,pl}+({Q}_{pi}-{Q}_{uf}){T}_{2,out}+{K}_{ip}({T}_{2,is}-{T}_{2,pl})\\ +\frac{{V}_{is}}{{V}_{ex}}{Q}_{uf}{T}_{2,is}+(\,-\,{b}_{1}{T}_{2,pl}{P}_{pl}+{b}_{2}P{T}_{2,pl}){V}_{pl},\end{array}\\ \frac{d({V}_{pl}P{T}_{2,pl})}{dt} & = & -{Q}_{pi}P{T}_{2,pl}+({Q}_{pi}-{Q}_{uf})P{T}_{2,out}+({b}_{1}{T}_{2,pl}{P}_{pl}-{b}_{2}P{T}_{2,pl}){V}_{pl},\\ \frac{d({V}_{pl}{D}_{pl})}{dt} & = & \begin{array}{c}-{Q}_{pi}{D}_{pl}+({Q}_{pi}-{Q}_{uf}){D}_{out}+{K}_{ip}({D}_{is}-{D}_{pl})\\ +\frac{{V}_{is}}{{V}_{ex}}{Q}_{uf}{D}_{is}+(\,-\,{d}_{1}{D}_{pl}{P}_{pl}+{d}_{2}P{D}_{pl}){V}_{pl},\end{array}\\ \frac{d({V}_{pl}P{D}_{pl})}{dt} & = & -{Q}_{pi}P{D}_{pl}+({Q}_{pi}-{Q}_{uf})P{D}_{out}+({d}_{1}{D}_{pl}{P}_{pl}-{d}_{2}P{D}_{pl}-\lambda {D}_{pl}){V}_{pl},\\ \frac{d({V}_{pl}{P}_{pl})}{dt} & = & \begin{array}{l}-{Q}_{pi}{P}_{pl}+({Q}_{pi}-{Q}_{uf}){P}_{out}+((\,-\,{a}_{1}{T}_{1,pl}{P}_{pl}+{a}_{2}P{T}_{1,pl})\\ +(\,-\,{b}_{1}{T}_{2,pl}{P}_{pl}+{b}_{2}P{T}_{2,pl})+(\,-\,{d}_{1}{D}_{pl}{P}_{pl}+{d}_{2}P{D}_{pl})){V}_{pl}.\end{array}\end{array}$$

**Table 2:** Nomenclature table with description of each symbol/parameter and respective units. For model parameters, corresponding values used in simulations are also mentioned.

Symbol	Description	Units	Value
*α*	Fraction of extracellular fluid volume in the interstitial compartment	—	—
*a*_1_/*b*_1_/*d*_1_	Association constant respectively for IS, pCS, and competitor drug in protein-solute dynamics	M^−1^min^−1^	10^8^
*a*_2_/*b*_2_/*d*_2_	Dissociation constant respectively for IS, pCS, and competitor drug in protein-solution dynamics	min^−1^	—
*A*	Blood flow area of fiber (inner cross-sectional area of fiber)	m^2^	3.5 × 10^−8^
*A*_d_	Effective flow area for dialysate around a fiber (annulus space between fibers)	m^2^	4.1 × 10^−8^
*C*_*pl/is/ic/d/tube*_	Concentration in plasma/interstitial pool/intracellular pool/dialysate side stream/tube, C ∈ {*T*_1_, *PT*_1_, *T*_2_, *PT*_2_, *D*, *PD*, *P*}	M	—
*C*_*in*_	Concentration of species in replacement fluid or in competitor drug infusion stream	M	—
*C*_out_	Concentration of species at dialyzer exit	M	—
*D*_*h*_	Dialyzer housing diameter	m	0.04
*D*_*pl*_	Competitor drug concentration in serum	M	—
*f*	Toxin free fraction	—	—
${G}_{{T}_{1}/{T}_{2}}$	T_1_/T_2_ generation rate	mg·min^−1^	—
*k*_1_	Association constant in *P*, *T*, and *PT* equilibrium (*a*_1_, *b*_1_, *d*_1_)	M^−1^min^−1^	—
*k*_2_	Dissociation constant in *P*, *T*, and *PT* equilibrium (*a*_2_, *b*_2_, *d*_2_)	min^−1^	—
${K}_{A,{T}_{1}/{T}_{2}/D}$	Equilibrium association constant for *T*_1_ or *T*_2_ or *D*	M^−1^	—
*K*_*ip*_	Free toxin/drug mass transfer coefficient between interstitial pool and plasma	mL·min^−1^	—
*K*_*ic*_	Free toxin/drug mass transfer coefficient between intracellular and interstitial pool	mL·min^−1^	—
${{\bf{K}}}_{{\bf{o}}}{\bf{A}}$	Dialyzer area mass-transfer coefficient	mL·min^−1^	600
*L*	Length of fiber	m	0.23
λ	Frist-order elimination rate constant for competitor drug	min^−1^	–
*N*	Number of fibers in dialyzer housing	—	12,300
*Pe*	Péclet number	—	—
*P*_*tot*_	Total protein concentration	M	—
*Q*_*b/d/p*_	Blood/dialysate/plasma flow rate	mL·min^−1^	—
*Q*_*pi*_	Plasma flow rate from the patient (in the extracorporeal circuit)	mL·min^−1^	—
*Q*_*R*_	HDF Replacement fluid flow rate or competitor drug infusion rate	mL·min^−1^	—
*Q*_*tube*_	Total flow rate in the tube-segment (after mixing with binding competitor or dilution fluid)	mL·min^−1^	—
*Q*_*uf*_	Ultrafiltration rate across the dialyzer	mL·min^−1^	10
*r*_*f*_	Hollow fiber inner radius	m	105 × 10^−6^
${\sigma }_{{C}_{p}}$	Reflection coefficient of concentration species in the serum	—	0.999 or 0
*t*_*f*_	Hollow fiber wall thickness	m	35 × 10^−6^
*Time*_*dia*_	Dialysis session duration	hr	4
*V*_*pl*/ *is*/ex/ *ic*_	Plasma/Interstitial/Extracellular/Intracellular fluid volume	L	—

*T_1_ denotes indoxyl sulfate (IS), *PT*_1_ albumin bound fraction of IS, *T*_2_ p-cresyl sulfate, *PT*_2_ albumin bound fraction of pCS, *D* is binding competitor drug, *PD* is albumin bound fraction of drug, and *P* denotes protein without toxins and drug.

The competition between IS, pCS, and a competitor drug for the same binding site on the protein molecule appears in the mass balance for free protein (Equation (2)). The λ in the free drug mass balance accounts for first order elimination of free drug by the liver. More details about drug metabolism are provided in section “*Competitor drug half-life*”. The mass balance for all species in the interstitial pool is given by Equation (3), which describes the free solute diffusion from intracellular to interstitial pool and from the interstitial to the plasma pool, as well as the ultrafiltration driven convective transfer of solutes from the interstitial to the plasma pool.

3 $$\begin{array}{rcl}\frac{d({V}_{is}{T}_{1,is})}{dt} & = & \begin{array}{c}{K}_{ic}({T}_{1,ic}-{T}_{1,is})-{K}_{ip}({T}_{1,is}-{T}_{1,pl})-\frac{{V}_{is}}{{V}_{ex}}{Q}_{uf}{T}_{1,is}\\ +(-{a}_{1}{T}_{1,is}{P}_{is}+{a}_{2}P{T}_{1,is}){V}_{is},\end{array}\\ \frac{d({V}_{is}P{T}_{1,is})}{dt} & = & ({a}_{1}{T}_{1,is}{P}_{is}-{a}_{2}P{T}_{1,is}){V}_{is},\\ \frac{d({V}_{is}{T}_{2,is})}{dt} & = & \begin{array}{c}{K}_{ic}({T}_{2,ic}-{T}_{2,is})-{K}_{ip}({T}_{2,is}-{T}_{2,pl})-\frac{{V}_{is}}{{V}_{ex}}{Q}_{uf}{T}_{2,is}\\ +(-{b}_{1}{T}_{2,is}{P}_{is}+{b}_{2}P{T}_{2,is}){V}_{is},\end{array}\\ \frac{d({V}_{is}P{T}_{2,is})}{dt} & = & ({b}_{1}{T}_{2,is}{P}_{is}-{b}_{2}P{T}_{2,is}){V}_{is},\\ \frac{d({V}_{is}{D}_{is})}{dt} & = & \begin{array}{c}{K}_{ic}({D}_{ic}-{D}_{is})-{K}_{ip}({D}_{is}-{D}_{pl})-\frac{{V}_{is}}{{V}_{ex}}{Q}_{uf}{D}_{is}\\ +(-{d}_{1}{D}_{is}{P}_{is}+{d}_{2}P{D}_{is}){V}_{is},\end{array}\\ \frac{d({V}_{is}P{D}_{is})}{dt} & = & ({d}_{1}{D}_{is}{P}_{is}-{d}_{2}P{D}_{is}){V}_{is},\\ \frac{d({V}_{is}P{D}_{is})}{dt} & = & \begin{array}{c}((-{a}_{1}{T}_{1,is}{P}_{is}+{a}_{2}P{T}_{1,is})+(-{b}_{1}{T}_{2,is}{P}_{is}+{b}_{2}P{T}_{2,is})\\ +(-{d}_{1}{D}_{is}{P}_{is}+{d}_{2}P{D}_{is})){V}_{is}.\end{array}\end{array}$$

The solute mass balances in the intracellular compartment (IC) are given by Equation (4), where toxin generation is assumed to occur at a constant rate and generated toxins diffuse into the interstitial compartment according to the concentration gradient. The generation rates for IS and pCS are 0.0248 mg/min and 0.0256 mg/min, respectively. The toxin generation rate is calculated such that pre-dialysis toxin concentration a week after on the same weekday matches with the current pre-dialysis toxin concentration, while intra-dialytic fluid removal is equal to inter-dialytic fluid gain. These generation rates are in accordance with literature reported values^13,34^. Albumin and albumin-bound solute fraction do not exist in the IC.

4 $$\begin{array}{ccc}\frac{d({V}_{ic}{T}_{1,ic})}{dt} & = & {G}_{{T}_{1}}-{K}_{ic}({T}_{1,ic}-{T}_{1,is}),\\ \frac{d({V}_{ic}{T}_{2,ic})}{dt} & = & {G}_{{T}_{2}}-{K}_{ic}({T}_{2,ic}-{T}_{2,is}),\\ \frac{d({V}_{ic}{D}_{ic})}{dt} & = & -{K}_{ic}({D}_{ic}-{D}_{is}).\end{array}$$

During dialysis, a patient loses a significant amount of fluid. It is assumed that fluid is removed from extracellular pool at a constant ultrafiltration rate (*Q*_*uf*_), where removal is proportional to the plasma and interstitial distribution volume^35^. Intracellular fluid volume is assumed to be constant during dialysis, as observed by multi-frequency bioimpedance^36^. Time dependent changes in plasma and interstitial fluid volumes are given by Equation (5).

5 $$\begin{array}{c}\frac{d{V}_{pl}}{dt}=-{Q}_{pi}+\,({Q}_{pi}-{Q}_{uf})+\alpha {Q}_{uf},\\ \frac{d{V}_{is}}{dt}=-\alpha {Q}_{uf},\,where\,\alpha =\frac{{V}_{is}}{{V}_{pl}+{V}_{is}}.\end{array}$$

#### Arterial tube-segment model (infusion site to dialyzer inlet)

Once blood (and solute within) leaves the patient, it enters the extracorporeal circuit. The competitor drug or replacement fluid in pre-dilution HDF is infused in this arterial tube segment at a constant rate during HD. The drug competes with toxins bound on albumin. In addition to drug-toxin competition on albumin, the model accounts for dilution introduced in the extracorporeal circuit either by drug infusion volume or by HDF replacement fluid. Owing to the dilution and/or competitor drug, the solutes, protein, and protein-solute complexes achieve a new equilibrium. We assumed that the infusion site is 0.5 m upstream of the dialyzer inlet. This tube-segment (highlighted in blue in Fig. 6) model is assumed to behave as plug-flow reactor model, where axial diffusion is neglected due to negligible diffusion coefficient of solutes in the direction of fluid flow^37^. Below is the generic sub-model explaining the shift in the dynamic protein-solute equilibrium between infusion site and dialyzer blood inlet (Equation (6)). The sub-model consists of 7 partial differential equations corresponding to concentration species mentioned in the Equation (6); an example describing spatiotemporal course of free IS in tube-segment is also provided.

6 $$\begin{array}{l}{Q}_{tube}={Q}_{pi}+{Q}_{R}\\ \frac{\partial {C}_{tube}}{\partial t}=-\frac{{Q}_{tube}}{{A}_{tube}}\frac{\partial {C}_{tube}}{\partial x}+Reaction\,Terms;C\in \{{T}_{1},P{T}_{1},\,{T}_{2},P{T}_{2},D,\,PD,\,P\}\\ e.g.,\,\frac{\partial {T}_{1,tube}}{\partial t}=-\frac{{Q}_{tube}}{{A}_{tube}}\frac{\partial {T}_{1,tube}}{\partial x}+(-{a}_{1}{T}_{1,tube}\cdot {P}_{tube}+{a}_{2}P{T}_{1,tube})\,.\end{array}$$

Here, ${Q}_{pi}$ is the plasma flow leaving the patient and entering the tube, ${Q}_{R}$ is the drug infusion volume or replacement fluid volume added at a constant rate in the arterial tube segment, ${Q}_{tube}={Q}_{pi}+{Q}_{R}$ is the flow rate in the tube-segment, *C*_tube_ denotes the substance concentration in the arterial tube-segment at any position and time during HD. For other symbols, please refer to the nomenclature Table 2. The initial and boundary condition for the arterial tube segment model are given in Equation (7).

7 $${C}_{x}(t=0)=0,\,{C}_{t}(x=0)=\frac{{Q}_{pi}{C}_{pl}+{Q}_{R}{C}_{in}}{{Q}_{tube}}$$

*C*_*pl*_ and *C*_*in*_ in Equation (7) denote the concentration of the species respectively in plasma and in infusion stream. In competitive binding, only the free drug exists in the infusion stream. Thus ${C}_{in}$ is zero for all other species, except for free drug, which competes with toxins bound to albumin, and increases toxins free fraction along the tube length and thus at the dialyzer inlet. In pre-dilution HDF, ${C}_{in}$ is zero for all species.

#### Dialyzer model (hollow fiber model)

The toxin laden blood combined with competitor drug or diluted by replacement fluid enters the dialyzer. It is assumed that blood distributes equally among all hollow fibers (*N*). The blood goes through the fiber lumen, where the dialysate flows in counter-current direction in interstitial space between fibers. Similar to blood flow, it is assumed that dialysate also distributes equally around all fibers. Along the fiber length, free IS, pCS, and free drug are removed by diffusion and convection. The solute diffusion is driven by the membrane mass-transfer coefficient (${{\bf{K}}}_{{\bf{o}}}{\bf{A}}$), while convection is driven by ultrafiltration or HDF replacement fluid removal. ${{\bf{K}}}_{{\bf{o}}}{\bf{A}}$ is a membrane property and depends on the molecular weight of the toxin, but remains fairly constant for molecules below 500 Da^38^. For the solutes IS, pCS, and infused drug, we used a ${{\bf{K}}}_{{\bf{o}}}{\bf{A}}$ of 600 mL/min^13^. A small amount of protein and protein-solute complexes are also removed by convection; their removal is impeded due to large membrane reflection coefficient (σ) for these large-sized molecules/complexes (σ = 0.999^39^). The reflection coefficient for small sized solutes is negligible (σ = 0)^39^, i.e. we assume there is no resistance for convective flux of small solutes by membrane pores tortuosity. A schematic of blood and dialysate flow along a single fiber is shown in Fig. 6. The flow profile for both plasma (mixed with drug stream or replacement fluid) and dialysate are given in Equation (8).

8 $$\begin{array}{l}{Q}_{tube}={Q}_{pi}+{Q}_{R}\\ {Q}_{p}(x)={Q}_{tube}-\frac{x}{L}({Q}_{uf}+{Q}_{R})\\ \,{Q}_{d}(x)={Q}_{di}+\frac{L-x}{L}({Q}_{uf}+{Q}_{R})\end{array}$$

It is assumed that the fluid removal rate along the dialyzer is uniform, i.e. plasma flow rate linearly decreases, while dialysate flow rate linearly increases. Mass balance in dialyzer results in the following generic model for the plasma and dialysate side (Equation (9)).

9 $$\begin{array}{l}\frac{\partial {C}_{p}}{\partial t}=-\frac{1}{N\cdot A}\frac{\partial ({Q}_{p}{C}_{p})}{\partial x}+\frac{1}{N\cdot A}\frac{\partial {Q}_{p}}{\partial x}{C}_{p}(1-{\sigma }_{{C}_{p}})-\frac{Pe}{{e}^{Pe}-1\,}\frac{1}{N\cdot A\cdot L}{{\rm{K}}}_{{\rm{o}}}{\rm{A}}\,({C}_{p}-{C}_{d})+Reaction\,Terms,\\ \,\frac{\partial {C}_{d}}{\partial t}=\frac{1}{N\cdot {A}_{d}}\frac{\partial ({Q}_{d}{C}_{d})}{\partial x}-\frac{1}{N\cdot {A}_{d}}\frac{\partial {Q}_{d}}{\partial x}{C}_{p}(1-{\sigma }_{{C}_{p}})+\frac{Pe}{{e}^{Pe}-1\,}\frac{1}{N\cdot {A}_{d}\cdot L}{{\rm{K}}}_{{\rm{o}}}{\rm{A}}\,({C}_{p}-{C}_{d})+Reaction\,Terms,\\ C\,\in \{{T}_{1},P{T}_{1},\,{T}_{2},P{T}_{2},\,D,\,PD,\,P\}\end{array}$$

The dialyzer model presented in Equation (9) accounts for the exchange of solutes in presence of both diffusion and convection. In the presence of convection (significant in HDF), the diffusive exchange of solutes is adjusted by a function of Péclet number (*Pe*) – defined as ratio of the convective mass transfer rate to the diffusive mass transfer rate^40^.

The patient model parameters were adapted from Maheshwari *et al*.^13^ The mass transfer rate of free solutes between plasma and interstitial pool (*K*_ip_) is 1135 mL/min and 100 mL/min between interstitial pool and intracellular pool (*K*_ic_). These mass transfer rates are the averages of point estimates for IS and pCS^13^. For binding competition during HD, we used tryptophan (2000 mg in 500 mL saline) and ibuprofen (800 mg in 200 mL saline); both drugs binds on the same binding site where IS and pCS bind on albumin^12^. In absence of literature source on competitor drugs’ inter-compartmental mass transfer rates, we assumed the same mass transfer rates that were used for IS and pCS. This assumption is physiologically reasonable since the molecular weight (surrogate of size) of toxins and drugs are comparable.

### Binding affinity of toxins and Competitor drug

Both IS and pCS are strongly bound to the albumin Sudlow drug binding site II^33^. For the assumed free fraction, 7% for IS and 5% for pCS^2^, the binding affinity of IS and pCS were calculated based on initial total concentrations of individual toxin and albumin.

10 $${K}_{A,{T}_{1}}=\frac{P{T}_{1}}{{T}_{1}\cdot P};{K}_{A,{T}_{2}}=\frac{P{T}_{2}}{{T}_{2}\cdot P};{K}_{A,D}=\frac{PD}{P\cdot D}\,$$

Here, *P* stands for initial free protein concentration. Note that $P={P}_{tot}-P{T}_{1}-P{T}_{2}-PD$, where ${P}_{tot}$ is the total protein concentration. The equilibrium association constant is also defined as *K*_*A*_ = *k*_1_*/k*_2_, where *k*_1_ and *k*_2_ are the association and dissociation constants. For IS, pCS, and competitor drug, the association constant (*a*_1_, *b*_1_, or *d*_1_) were assumed to be 10^8^ M^−1^min^−1^ ^41^; the dissociation constant was calculated using the above definition of *K*_*A*_ of the solute under consideration. The binding affinities for tryptophan and ibuprofen are 1.73 × 10^4^ M^−1^ and 1.76 × 10^5^ M^−1^, respectively^42,43^. Both tryptophan and ibuprofen prescriptions are within FDA approved dosage guidelines. The added drug infusion volume was removed by adjusting the ultrafiltration rate (see Equation (8)).

### Competitor drug half-life

Tryptophan and ibuprofen, the competitor drugs considered in analysis, are continuously metabolized by liver. The reported half-life of tryptophan and ibuprofen is 2.83 hrs and 2 hrs, respectively^44,45^. The drug half-life, stated here, corresponds to total serum concentration, i.e. after one half-life, the total serum concentration will be half of the peak serum concentration. Note that this total concentration comprises free and protein-bound drug fraction, and only free fraction is metabolized which leads to further release of free drug from albumin binding site. To account for this physiological aspect in our model, we adjusted the free drug half-life such that it conforms to the standard definition of drug half-life. For the mentioned binding affinities for tryptophan and ibuprofen 1.73 × 10^4^ M^−1^ and 1.76 × 10^5^ M^−1^, respectively, the calculated half-life of free tryptophan is 6.24 min and of free ibuprofen is 1 min. In this calculation, it is assumed that peak serum concentration of free drug is in equilibrium with free drug concentration in the interstitial and intracellular pool.

### Hemodiafiltration

We simulated both pre- and post-dilution HDF. The blood and dialysate flow profiles for pre-dilution HDF are shown in Equation (8). For post-dilution HDF, the arterial tube-segment model shown in Fig. 6 is replaced by a similar venous tube-segment model (model not shown), which receives dialyzer blood output and post-dilution replacement fluid to compensate for excess fluid removed along the dialyzer length. The output of the venous tube segment is delivered to the patient as venous return. Unlike post-dilution HDF where replacement fluid volume cannot be too large due to hemoconcentration in the dialyzer, the pre-dilution HDF can run with much higher replacement fluid volume. We tested two pre-dilution HDF scenario with a replacement fluid volume of 20 L and 60 L, and a post-dilution HDF scenario with 20 L replacement fluid volume^9^.

### Membrane adsorption

Membrane adsorption works such that free toxin is adsorbed on the membrane surface which maintains a high concentration gradient between blood and dialysate stream. In ideal conditions, this technique can be labeled equivalent to an infinite dialysate flow rate, i.e. toxin concentration in dialysate side is zero. In this work, we did not attempt to model the toxin adsorption kinetics in the membrane; rather we simulated the adsorptive removal of PBUTs such that during conventional HD, concentration of free toxin in the dialysate side is always zero, equivalent to hypothetical ideal membrane adsorption.

All dialysis modalities were simulated for a dialysis duration of 4 hours (*Time*_*dia*_). The initial IS and pCS concentrations were set to 100 µM (21.3 mg/L; 93% bound) and 150 µM (28.2 mg/L; 95% bound), respectively; total serum albumin concentration was 600 µM (≈ 4 g/dL). The blood flow rate (*Q*_*b*_) was 300 mL/min, dialysate flow rate (*Q*_*d*_) was 800 mL/min, and ultrafiltration volume was 2.4 L. For each modality, we calculated the toxin reduction ratio (RR, %), net toxin removal (mg), and toxin clearance (mL/min) using the expressions given in Equation (11).

11 $$\begin{array}{l}RR( \% )=(1-\frac{{C}_{pl,t=240}}{{C}_{pl,t=0}})\times 100\\ Net\,Removal=\,{[{C}_{pl}{V}_{pl}+{C}_{is}{V}_{is}+{C}_{ic}{V}_{ic}]}_{t=0}-\,{[{C}_{pl}{V}_{pl}+{C}_{is}{V}_{is}+{C}_{ic}{V}_{ic}]}_{t=240}+G\times Tim{e}_{dia}\,\\ Clearance=\frac{Net\,Removal}{(\frac{{C}_{pl,t=0}-{C}_{pl,t=240}}{\mathrm{ln}\,{C}_{pl,t=0}-\,\mathrm{ln}\,{C}_{pl,t=240}})}\,;C=Total\,toxin\,concentation\,\end{array}$$
